# The Importance of Natural Antioxidants in Female Reproduction

**DOI:** 10.3390/antiox12040907

**Published:** 2023-04-11

**Authors:** Janka Vašková, Zuzana Klepcová, Ivana Špaková, Peter Urdzík, Jana Štofilová, Izabela Bertková, Marek Kľoc, Miroslava Rabajdová

**Affiliations:** 1 Department of Medical and Clinical Biochemistry, Faculty of Medicine, Pavol Jozef Šafárik University in Košice, Trieda SNP 1, 040 11 Košice, Slovakia; janka.vaskova@upjs.sk (J.V.);; 2 Medirex, a.s., Holubyho 35, 902 01 Pezinok, Slovakia; 3 Department of Gynaecology and Obstetrics, Faculty of Medicine, Pavol Jozef Šafárik University in Košice, Trieda SNP 1, 040 11 Košice, Slovakia; 4 Center for Clinical and Preclinical Research MEDIPARK, Department of Experimental Medicine, Faculty of Medicine, Pavol Jozef Šafárik University in Košice, Trieda SNP 1, 040 11 Košice, Slovakia

**Keywords:** vitamin A, vitamin E, vitamin C, vitamin B9, melatonin, L-carnitine, resveratrol, quercetin, antioxidants, Nrf2, NF-κB, fertility

## Abstract

Oxidative stress (OS) has an important role in female reproduction, whether it is ovulation, endometrium decidualization, menstruation, oocyte fertilization, or development andimplantation of an embryo in the uterus. The menstrual cycle is regulated by the physiological concentration of reactive forms of oxygen and nitrogen as redox signal molecules, which trigger and regulate the length of individual phases of the menstrual cycle. It has been suggested that the decline in female fertility is modulated by pathological OS. The pathological excess of OS compared to antioxidants triggers many disorders of female reproduction which could lead to gynecological diseases and to infertility. Therefore, antioxidants are crucial for proper female reproductive function. They play a part in the metabolism of oocytes; in endometrium maturation via the activation of antioxidant signaling pathways Nrf2 and NF-κB; and in the hormonal regulation of vascular action. Antioxidants can directly scavenge radicals and act as a cofactor of highly valuable enzymes of cell differentiation and development, or enhance the activity of antioxidant enzymes. Compensation for low levels of antioxidants through their supplementation can improve fertility. This review considers the role of selected vitamins, flavonoids, peptides, and trace elements with antioxidant effects in female reproduction mechanisms.

## 1. Introduction: Infertility Nowadays

Oxidative stress affects many physiological processes, not only those involved in the reproductive system. Low levels of reactive oxygen and nitrogen species (RNOS) have a significant signaling role in the normal functioning of the ovaries (ovulation) and the endometrium of the uterus (decidualization, healing after the menstrual phase without scarring), and thus in fertility; stimulate energy production and angiogenesis; and regulate the inflammatory response in the female menstrual cycle. Chronic disruption of the physiological redox signaling activities necessary for the proper functioning of the female reproductive system can lead to the development and progression of gynecological, immunological, and hormonal disorders that lead to subfertility and infertility [1,2,3]. Infertility is a reproductive disorder defined as the inability to conceive after more than one year of unprotected intercourse [4] and affects 48 million couples worldwide [5] nowadays. Statistical data indicate that both male and female infertility account for approximately 45% [6] of infertility, and future prediction models show an even lower rate of new births [7,8]. Common infertility treatment methods include the surgical removal of defective tissue and hormonal supplementation [9] which are invasive methods used to “adjust” the receptive endometrium of the mother to allow the successful implantation of a competent embryo at the optimal stage of development as a key aspect of assisted reproduction technology (ART) [10]. The success rate of a single in vitro fertilization (IVF) cycle is circa 30% [11] and this figure has not improved over the past decade [12]. One of the main tools for improving the female fertility rate is to decrease/normalize OS conditions to the physiologically desirable level and create a reproductive-friendly microenvironment via the right lifestyle and supplementation of antioxidants.

## 2. Female Reproduction Regulated by Reactive Oxygen and Nitrogen Species

Fertility is a complex process that comprises the decidualization of the uterine endometrium which is essential for the establishment of a successful pregnancy and which occurs in response to elevated levels of the ovarian hormones (estrogen—E2 and progesterone—P4) regulating the hormonal changes required for embryo implantation during the secretory phase of the menstrual cycle [13]. This process occurs under specific physiological levels of RNOS related to the initiation of signals for interaction in the decidualized uterine endometrium, which is later exposed to extensive changes in oxygen tension during fertilization and pregnancy [14]. Undoubtedly, maintaining the balance between oxidants and antioxidants is important for the proper functioning of uterine physiological processes. Oxidative stress in general is a result of an imbalance between antioxidant capacity and the production of pro-oxidants such as reactive oxygen species (ROS) and reactive nitrogen species (NOS) [14], but OS may significantly impact the normal female reproductive lifespan. Ovulation, oocyte maturation, ovarian steroidogenesis [15], luteolysis, luteal maintenance in pregnancy [16], the development of follicles and blastocysts, and blastocyst implantation as well as embryo development [17] are regulated via RNOS which fulfill the role of signaling. The RNOS signaling pathways involved in female reproduction such as mTOR, NF-κB, Nfr2/Keap1/ARE, FOXO, or MAPK/ERK, are affected by the level of RNOS [16]. The NF-κB signaling pathway consists of 5 transcription factors, namely RelA (p65), RelB, c-Rel, NF-κB1 (p105/p50), and NF-κB2 (p100/p52) [18]. This pathway is regulated via inhibitors of κB (IκB) which are phosphorylated by IκB kinases (IKKs) and degraded by the proteasome [19]. The NF-κB dimers enter the nucleus and as transcription factor downstream pro-inflammatory genes (TNFα, MIF, MMPs), cytokines (IL1/6/8), cell migration, and invasion genes (VCAM1, ICAM1, HCAM), as well as proliferation genes (WIAP, Bcl-2, Bcl-Xl) [18]. The activity of NF-κB is elevated by estrogen binding to its receptors activating several pro-inflammatory pathways such as CXL12/CXCR4, PI3K/Akt, or MAPK/ERK [11,14]. The overproduction of ROS is an important inducer of chronic NF-κB-mediated inflammatory responses [20] which can trigger cells into pathologies. Under physiological conditions, the NF-κB subunit p65 and progesterone receptor are mutually repressed [21]. Progesterone inhibits the NF-κB-induced pro-inflammatory factors in endometriotic cells [18,21]. High mobility group box-1 (HMGB-1) activates NF-κB via binding to its TLR4 (Toll-like receptor 4) receptor and induces inflammatory responses with sustained OS [22]. The HMGB-1/TLR4/NF-κB axis can induce the production of pro-oxidants (iNOS, NO), decrease the activities of SOD, GPx, CAT, and activate the phosphorylation of IKKs and the proliferation and invasion of cells [18].

The endogenous ROS signal originates from inflammatory signals from macrophages and neutrophils which affect endometrial breakdown and repairment [23]. Progesterone withdrawal through ROS-induction stimulates the activity of NF-κB/COX2 signaling as well as cytokines (IL1, IL6, IL8, TNF-α, etc.) and matrix metalloproteinases (MMPs) activation [23] resulting in an influx of leukocytes into the uterus and leading into the endometrial breakdown and shedding [16].

An increased ROS activates the nuclear factor E2-related factor 2 (Nrf2) transcription factor which binds to the antioxidant response element (ARE) in the promoter of the target genes to induce the expression of antioxidant genes such as NAD(P)H:quinone oxidoreductase 1, glutathione synthesis and the genes involved in mitochondrial quality and quantity control [15,16,17,18]. The Nrf2 under physiological ROS levels is bound to Keap1, is constantly ubiquitinated via Cul3 E3 ubiquitin ligase, and is subsequently degraded by the proteasome [24]. In the case of elevated ROS, Keap1 is disconnected from Nrf2 and Nrf2 is translocated to the nucleus, where it is heterodimerized with sMaf and binds to the ARE which activates the transcription of its target genes [25].

**Figure 1:**
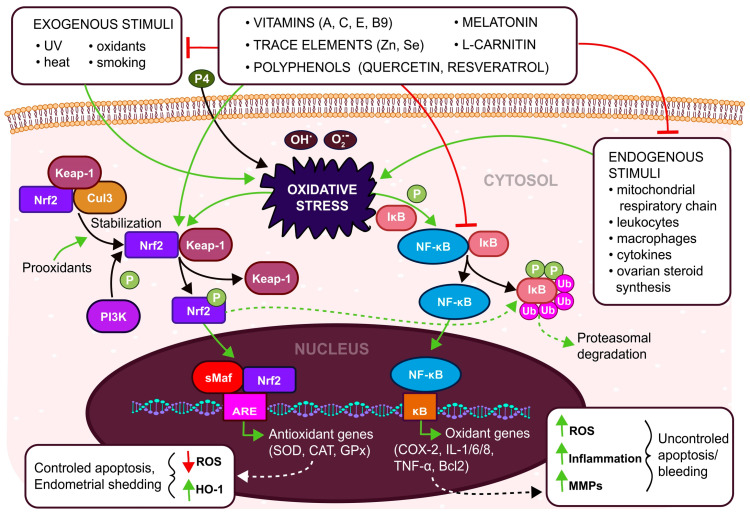
Oxidative stress (OS) is a stimulant (green arrows) of two main antioxidant signaling pathways-Nrf2 and NF-κB. Nrf2 signaling is involved in the expression of antioxidant enzymes, minimizing the effect of OS. NF-κB signaling is involved in the expression of genes for cells surviving under OS. Exogenic antioxidants (vitamins, trace elements, polyphenols, etc.) activate Nrf2 signaling and inhibit (red lines) the NF-κB pathway and eliminate exogenous as well as endogenous stimuli of OS. (Nrf2—Nuclear factor erythroid 2-related factor 2, Keap-1—Kelch-like ECH-associated Protein 1, Cul3—cullin protein 3, PI3K—Phosphoinositide 3-kinase, P—phosphate, NF-κB—Nuclear factor kappa-light-chain-enhancer of activated B cells, IκB—inhibitor of nuclear factor kappa B, sMaf—musculoaponeurotic fibrosarcoma, ARE—Antioxidant responsive element, κB—κB DNA binding, SOD—Superoxide dismutase, CAT—Catalase, GPx—Glutathione peroxidase, COX-2—Cyclooxygenase 2, IL-1/6/8—Interleutkin 1/6/8, TNF-α—Tumor necrosis factor α, Bcl2—B-cell lymphoma-2, ROS—reactive Oxygen Species, HO-1—Heme Oxygenase 1, MMPs—Matrix metalloproteinases, Ub—ubiquitin).

On the other hand, in OS conditions (Figure 1) ROS has a role in the regulation of NF-κB signaling as well as Nrf2 signaling, resulting in abnormal bleeding and uncontrolled apoptosis [14,21,26] in pathological processes and diseases such as endometriosis, polycystic ovary syndrome (PCOS), and tubal and idiopathic infertility [27,28,29]. During pregnancy, increased placental OS can trigger pregnancy-related problems such as pre-eclampsia, intrauterine growth restrictions, gestational diabetes mellitus, or premature birth [30]. Elevated ROS initiates mitochondrial dysfunction and imbalance in the hormonal function of women [27]. Increased stress can stimulate the over-secretion of cortisol which can suppress the gonadotropin-releasing hormone (GnRH), luteinizing hormone (LH) and follicular stimulating hormone (FSH) [31]. Suppressing the production of estradiol leads to the failure of oocyte maturation, resulting in infertility [24]. The ROS of respiratory chain complexes is physiologically generated mainly via complexes I, III, and IV [32]. In oocytes, ROS starts to build up as soon as complex I is assembled [33]. The dysfunction of respiratory complex I or overproduction of ROS leads to oocyte maturation failure.

## 3. Antioxidant Systems in Female Reproduction

The human body has developed a variety of enzymatic and non-enzymatic antioxidant systems available to combat OS [17]. Enzymatic antioxidant protection consists of key enzymes, namely catalase (CAT), glutathione peroxidase (GPX), glutathione reductase (GSR), and superoxide dismutase (SOD), which are important in the prevention of lipid peroxidation and the maintenance of the proper function of cell membranes [34]. The total level of SOD (Cu/Zn/Mn-SOD) increases in the endometrium from the proliferative phase to the mid-secretory phase and decreases in the late-secretory phase, and on the contrary, lipid peroxidation increases as a physiological action of ROS [23]. Non-enzymatic antioxidants, mainly taken in food or endogenous biosynthesis, include polyphenols (quercetin, resveratrol, baicalin), carotenoids (β-carotene, lycopene, lutein), low-molecular-weight antioxidants (glutathione, uric acid), trace elements (zinc and selenium), vitamins (A, E, C, B9), and others (L-carnitine, melatonin, acetylcysteine) [35,36]. The level of endogenous antioxidants is mostly stable and these molecules are only activated as necessary [37]. The exogenous antioxidants are received in food so their levels fluctuate based on diet [38]. This review focuses on selected non-enzymatic antioxidants in connection with the signaling pathways involved in female reproduction disorders.

Most antioxidants, in addition to their ability to scavenge radicals, activate Nrf2 and inhibit NF-κB pathways [11,26]. There is evidence that some antioxidants which naturally occurring in diet may have benefits for the female reproductive system. A list of selected antioxidants is shown in Table 1.

### 3.1. Carotenoids, Ascorbic Acid, Tocopherol, and Folic Acid Affect the Female Reproductive System

Understanding the mechanism of action of vitamin A, C, E, and B9 represents a milestone in the study of antioxidant properties [71]. Serum concentrations of antioxidants are associated with steroidogenesis and further elucidate the potential role of antioxidants in women’s reproductive health due to the protection the cell compartments from oxidative damage and regulation the physiological development of uterine and ovarian cells across a normal menstrual cycle [72].

Vitamin E exists in eight isoforms, from which the α-tocopherol is the most effective form and γ-tocopherol is the most common [73]. This naturally occurring fat-soluble vitamin acts as a scavenger of peroxyl radicals in cell membranes (affects the stability of the membrane and indirectly modulates the signaling properties of membrane proteins) and terminates lipid peroxidation chain reactions [73]. After the reaction with lipid peroxyl radical, a stable tocopherol radical is formed which undergoes reverse conversion to α-tocopherol ensured by ascorbic acid or glutathione GSH [74].

**Figure 2:**
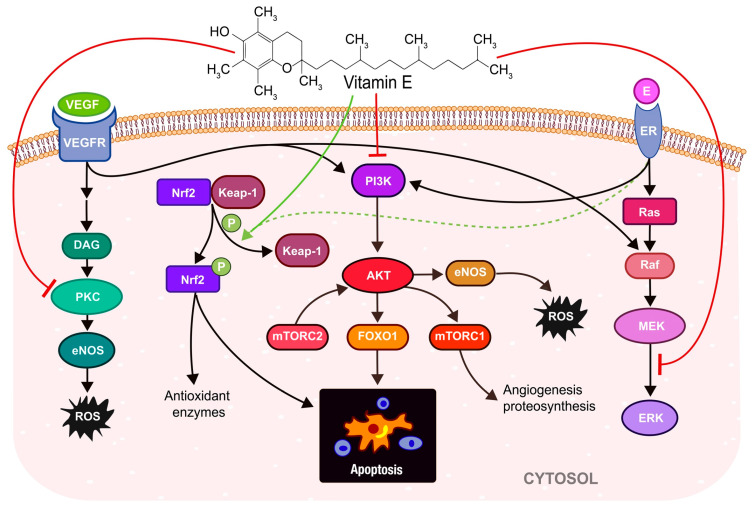
Vitamin E (tocopherol) action in response to oxidative stress. It can stimulate Nrf2 (Nuclear factor erythroid 2–related factor 2) signaling (green arrow) and cell apoptosis. Tocopherol can affect signaling of growth factors (Vascular Endothelial Growth factor, VEGF) and hormones (estrogen, E) by inhibition of PKC (Protein kinase C) (red line), which activates protein phosphatase, and dephophorylates PKC and inhibits its activity, which in turn leads to lower ROS mediated via uncoupled eNOS. Tocopherol also suppress E-dependent ERK activation (red line) via its ability to bind to the estrogen receptor (ER). On the other hand, vitamin E can inhibit apoptosis through the PI3K/AKT/mTOR pathway (red line). (VEGFR—Vascularendothelial Growth Factor Receptor, DAG—Diacylglycerol, Keap-1—Kelch-like ECH-associated Protein 1, P—phosphate, PI3K—Phosphoinositide 3-kinase, AKT—Protein kinase B (PKB) also known as AKT, FOXO1—Forkhead box protein O1, mTORC1—mammalian Target of Rapamycin Complex 1, mTORC2—mammalian Target of Rapamycin Complex 2, eNOS—endothelial NOS, Ras—Rat sarcoma virus, Raf—Rapidly accelerated fibrosarcoma, MEK—Mitogen-activated protein kinase, ERK—Extracellular signal-regulated kinase).

Vitamin E is involved in the alter expression of transcription factors to significantly decrease oxidative stress [73]. The AP1 (activator protein-1) transcription factor family, and the promoter regulatory element ARE, are involved in the regulation of redox homeostasis that can affect the NF-κB, MAPK/ERK/PI3K, and Nrf2 pathways (Figure 2) and modulate gene expression, including certain proteins that control cell cycle progression due to the reduction of PKC (protein kinase C), leading to decreased activation of MAPK/ERK (targeting cyclin D/E1, p27/53) and inflammatory response via inhibition of COX2, and they also regulate angiogenesis through the modulation of VEGF [73].

Vitamin E with its antioxidant properties has been reported to be efficient in reproductive- and pregnancy-related disorders [41,44]. As an antioxidant, it has benefits regarding reproductive diseases via improving endometrial thickness which is favorable in women with implantation failure [75]. Vitamin E in combination with other supplements such as zinc, selenium, iron, and L-arginine could increase the ovulation and pregnancy rate [41,47]. In addition to its antioxidant properties, it also has anti-inflammatory effects in reducing the production of prostaglandins [76]. Vitamin E regulates angiogenesis as well, as proved by significantly reduced VEGF in serum in women with PCOS [77].

Ascorbic acid, better known as water-soluble vitamin C, is a part of the ascorbate peroxidases-glutathione reductase (APXs-GR) antioxidant system that can scavenge superoxide (O_2_^•−^) and hydroxyl (^•^OH) radicals, resulting in the formation of ascorbate radicals which act more effectively than the CAT or SOD antioxidant system [78]. Among its antioxidant properties, it fulfils its role in collagen synthesis, vasculogenesis, aging, cell proliferation, and differentiation [79]. From the two forms of vitamin C, the reduced form (ascorbic acid,) and the oxidized form (dehydroascorbic acid), only its reduced form has antioxidant capacity [36,80]. Administration of vitamin C to women with a luteal phase defect increased the level of progesterone in serum [81] and the pregnancy rate [78]. Ascorbic acid can compensate significantly for the adverse effects of aging in the ovary as was shown in mice [79], probably via inhibition of senescence through suppression of ROS production and AKT/mTOR signaling in mesenchymal stem cells [82]. The protective effects of vitamin C after exposure to oxidative damage lead to suppressed apoptosis by reducing the expression of caspase-3 or -8 in the ovarian and uterine tissues of treated rats and a decrease in the level of anti-mullerian hormone (AMH), the marker for granulosa cells [83]. However, ascorbic acid also has a pro-oxidative effect which is associated with the interaction with transition metal ions such as iron and copper [84] and can produce O_2_^•−^ in the oxidation of the metal. Many studies were found regarding the antioxidant effect of vitamin E in combination with vitamin C in women of reproductive age with increased oxidative stress [52]. These two vitamins are promising in the prevention of pre-eclampsia and preterm birth [85], and contribute effectively to the reduction of pain in women with endometriosis [86,87].

**Figure 3:**
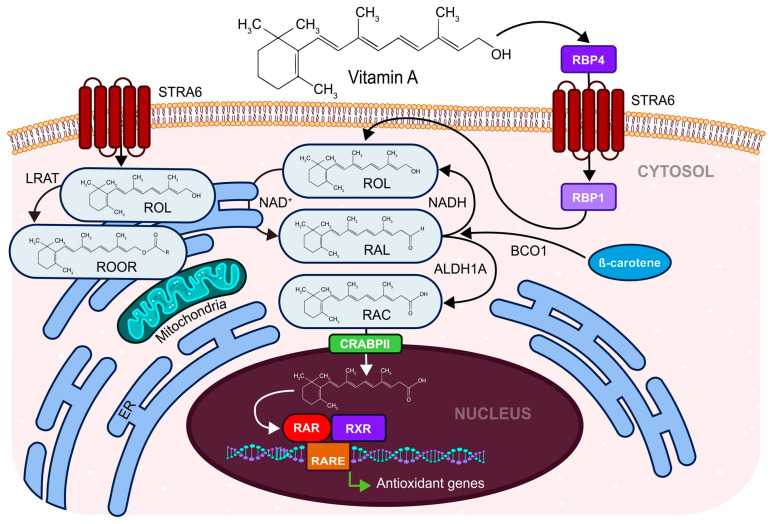
Vitamin A (retinol) activity in response to oxidative stress. Vitamin A is carried on RBP4 (Retinol Bindin Protein 4) protein and is translocated to the cell cytosol via its integral receptor STAR6 (Stimulated by Retinoic Acid 6 Receptor). In the cytosol, vitamin A is carried on RBP1 (Retinol Binding Protein 1). Vitamin A (retinol, ROL) undergoes oxidation on the endoplasmatic reticulum (ER) to form RAL (retinal) and, via catalytic activity of ALDH1A (aldehyde dehydrogenase member A1 or retinaldehyde dehydrogenase 1), to form RAC (retinoic acid). The β-carotene can also be converted into RAL by β-carotene 15–15′-oxygenase. RAC enters the nucleus and binds to regulative elements on the DNA and affects the target antioxidant gene transcription. ROL can be converted via the catalytic activity of LRAT (Lecithin Retinol Acyltransferase) to its esters (ROOR) as well.

The antioxidant effects of carotenoid, fat-soluble vitamin A, consist of the absorption of peroxyl radicals and singlet oxygen [88]. Vitamin A was shown to be essential in several physiological processes, especially in reproduction, the immune system via an inflammatory response as it is related to the expression of cyclooxygenases, the generation of nitric oxide, prostaglandins [89], cell differentiation, vision, and bone metabolism [55,56]. Vitamin A participates in signaling during embryogenesis [57] via the induction of activation of retinoic acid receptor (RAR) and retinoid X receptor (RXR) transcription factors and their binding to RARE sites in DNA and also activates the expression of developmental genes (Figure 3) [90]. This positive effect on embryo development was confirmed by a study of the addition of retinol to the blastocyst’s culture media which improved the development of rabbit embryos [58].

The importance of retinoids in embryo development is via key embryonic signaling pathways such as Wnt, HH, FGF, and TGF. During the 3rd trimester of pregnancy, the demand for vitamin A increases [57]. Its deficiency is associated with an increased risk of premature birth and maternal anemia, and it is an important determinant for early lung development and the formation of alveoli in utero [91]. Further studies suggest that the detection of vitamin A and E in follicular fluid has a promising association with the status of the developing embryo, and therefore it could enable the selection of a competent embryo for transfer [92] during the IVF process.

Folate, a water-soluble vitamin B9, is a crucial coenzyme in transferring the methyl group in the so-called single-carbon cycle [93] and is necessary for the formation of red and white blood cells, nucleotide biosynthesis, DNA reparation, and the metabolism of amino acids [94]. Folate is comparable to vitamins C and E, and can also act as an effective antioxidant of reproductive health in vivo [95]. In female reproduction, folate is also important for oocyte quality and maturation, implantation, embryogenesis, placentation, fetal growth, and organ development during pregnancy [96]. Folate administration is related to the prevention of pregnancy-related disorders such as intrauterine growth retardation, neural tube defects, anencephaly, spina bifida, increased premature births, and miscarriages [45]. The oral administration of folate can reduce the number of immature oocytes in PCOS women undergoing IVF therapy [97]. In addition, concentration folate in follicular fluid significantly affects the pregnancy rate [98].

**Figure 4:**
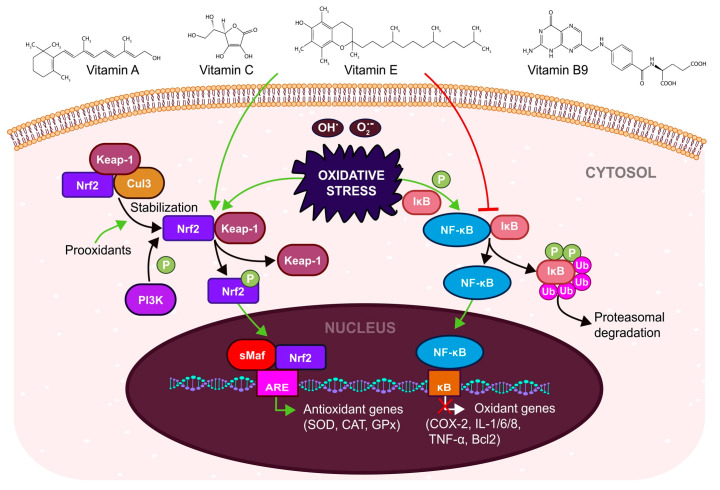
A summary of vitamin A-C-E-B9 action in the activation (green arrows) of Nrf2 signaling and suppression (red line) of NF-κB signaling. (Nrf2—Nuclear factor erythroid 2–related factor 2, Keap-1—Kelch-like ECH—associated Protein 1, Cul3—cullin protein 3, P—phosphate, NF-κB—Nuclear factor kappa-light-chain-enhancer of activated B cells, IκB—inhibitor of nuclear factor kappa B, sMaf—musculoaponeurotic fibrosarcoma, ARE—Antioxidant responsive element, κB—κB DNA binding, SOD—Superoxide dismutase, CAT—Catalase, GPx—Glutathione peroxidase, COX-2—Cyclooxygenase 2, IL-1/6/8—Interleutkin 1/6/8, TNF-α—Tumor necrosis factor α, Bcl2—B-cell lymphoma-2).

A summary of the antioxidant action of vitamins via regulation of Nrf2 and NF-κB signaling is showed in Figure 4.

### 3.2. L-carnitine Ensures the Physiological Need for ATP in Women’s Reproduction Processes

L-carnitine (LC) and its acetylated form acetyl-L-carnitine (ALC) are naturally occurring derivates of lysine and methionine amino acids. They play an important role in intermediate metabolism as they act as a cofactor in the transport of free fatty acids from the cytosol to the mitochondrial matrix [66,67,68]. The supplementation of L-carnitine in oocytes and embryos increases energy production by β-oxidation of fatty acids, scavenges ROS, has anti-apoptotic and anti-inflammatory effects, and results in an increased pregnancy rate, as well as enhanced cryo-tolerance [99]. L-carnitine is converted to ALC in the mitochondria. ALC regulates the ratio of acetyl-CoA/CoA to maintain glucose metabolism via Krebs’ cycle and increases the utilization of pyruvate in gluconeogenesis [67]. Furthermore, LC can increase levels of vitamin C, vitamin E, and antioxidant enzymes such as CAT and SOD [100], and can prevent ROS formation in the respiratory chain via control of the transport of long-chain fatty acids (FA) to the mitochondria to facilitate their utilization by β-oxidation [101]. Moreover, LC is involved in decreasing apoptosis by the inhibition of TNF-α, IFN-γ, and IL-2/4/6, and in increasing the production of PGE1/2 which induces cytokine release and thus uterine defects such as endometriosis, and maintains their apoptic and inflammatory properties [101]. LC and ALC affect the hypothalamic-pituitary-gonadal axis to promote the secretion of GnRH from the hypothalamus by increasing blood levels of estradiol, progesterone, and LH and, on the contrary, by decreasing the blood level of prolactin [102]. Due to its ability to improve the hormonal profile and metabolic parameters of women with reproductive disorders, the administration of LC to patients with functional hypothalamic amenorrhea improved the course of the disease [103] and reduced cytokine production. It also improved the hormonal profile of patients with endometriosis [17]. In women with PCOS, L-carnitine has a beneficial effect on ovulation and pregnancy rates, leads to improved endometrial thickness, and increases the serum estradiol level [104]. The reproductive potential (number of oocytes and viable embryos) in women undergoing IVF treatment [105] positively correlates with a high value of LC in the blood serum as well as in the follicular fluid [106]. The addition of L-carnitine to the culture medium improved oocyte and embryo quality as it is associated with β-oxidation of FA which is capable of producing an enormous amount of ATP which is essential for oocyte maturation and early embryo development [69,73,74,107].

### 3.3. Melatonin Affects the Normal Hormonal Development and Functioning of the Female Reproductive Organs

Melatonin (N-acetyl-5-methoxytryptamine) is a naturally occurring peptide hormone secreted by the pineal gland and multiple extra-pineal tissues (e.g., uterus, ovaries, and placenta) involved in the regulation of response to darkness, immune responses, inflammation, and microenvironmental angiogenesis [108]. Therefore, it is involved in the regulation of the sleep cycle and in multiple biological functions such as the metabolism of lipids, saccharides and the immune response [109]. Melatonin was shown to decrease OS, inflammation processes, and apoptosis [108]. It is directly involved in the detoxification of ROS and NOS, and is indirectly involved in the stimulation of enzymatic antioxidants and the suppression of pro-oxidants [110]. Melatonin was shown to downregulate COX-2 protein levels and upregulate SOD, GPx, CAT, and Bcl-2 activity, possibly via upregulating the Nrf2 signaling pathway [108]. Melatonin has an important role in female reproduction during childhood and puberty when it controls women’s reproductive endocrinological system [111] and affects the ovulation process [108]. Melatonin levels and fertility both decrease with age, [111] and these two processes could be linked. Upregulated E2 causes P4 resistance which leads to reproductive disorders such as endometriosis or PCOS [112]. Melatonin suppresses E2 production from the pre-ovulatory follicle via MAPKs, suppresses aromatase activity, and decreases the level of ERα in ovaries and the level of ERβ in uterine tissue [108]. Some studies state that the concentration of melatonin is positively correlated with the number of follicles and follicle size in women undergoing IVF therapy and is also positively correlated with IVF outcome [113,114]. A decreased concentration of melatonin in the follicular fluid could be responsible for anovulation and poor quality oocytes in a patient with PCOS and, therefore, melatonin treatment can improve the quality of oocytes during follicular maturation [114]. Due to the antioxidant properties of melatonin, it plays an important role in neovascularization and angiogenesis via decreasing Ang-1/2, VEGF, and VEGFR expression during hypoxia. Due to its anti-angiogenic activity, it can reduce endometrial implant volumes in patients with endometriosis [108]. Melatonin can also inactivate MMPs (mainly MMP3 and MMP9), something which could also be beneficial in a patient with endometriosis [108]. Melatonin and its receptors MT1/2 are constantly expressed by placental cells to promote survival and syncytialization of the cytotrophoblast while reducing ROS and angiogenesis [112]. Melatonin supplementation for women with pre-eclampsia-related OS limiting hypertension [112] and patients with unexplained infertility can increase the pregnancy rate and embryo quality [30,31,115] via an upstream antioxidant Nrf2 activity and downstream activity of NF-κB [116,117].

### 3.4. Polyphenols—Quercetin and Resveratrol Have a Dual Function in the OS of the Female Reproductive System

We would emphasize the importance of micronutrients in the diet, especially polyphenols, in the prevention and treatment of a wide range of chronic inflammatory diseases that can affect human fertility. Flavonoids are the most studied group of polyphenols, and are formed by two aromatic rings bound by three carbon atoms which form a heterocycle [118]. The most common flavonoids include quercetin, myricetin, catechin, and others [119]. Quercetin (3,3′,4′,5,7-pentahydroxyflavone) has anti-inflammatory, antiproliferative, and antioxidant properties [120,121]. Quercetin does not harm healthy cells [122] and could be an ideal agent for antioxidant treatment (it can reduce ROS via donating electrons) and is an inhibitor of cell growth and the cell cycle via various mechanisms [120,122,123] which is possible due to its antioxidant as well as prooxidant properties. Quercetin as an apoptotic inducer (at its high concentration) activates p53 and induces elevation of pro-apoptotic BAX, caspase-3/9, and also activates a reduction in anti-apoptotic Bcl-2 and survivin [122]. This polyphenol can enhance ARE binding activity and Nrf-2-mediated transcription activity to induce antioxidant enzyme expression [124] such as SOD, GPx, CAT, and GR [125] which protect against OS and inflammatory responses via inhibition of the NF-κB pathway [126].

Oocytes cultured in a medium supplemented with quercetin showed better in vitro maturation and early embryonic development ability. The higher quality of the oocytes, increased the oocyte fertilization rate as well as the blastocyst-formation rate, and resulted in a higher number of high-quality blastocysts [127]. On the other hand, quercetin has shown an ability to inhibit the activity of transglutaminase 2 (TG2) which is present in the endometrial epithelium during embryo implantation [128]. Therefore, long-term use of quercetin as a supplement may have a mild adverse effect on female fertility [128]. It is known that quercetin, as a phytoestrogen, has a pleiotropic effect on the uterus as it has an estrogenic effect as well as an anti-estrogenic effect. At high doses it may pose a potential risk and trigger neoplastic changes in the uterus [62] as it has prooxidative stimuli and apoptotic responses.

One of the best-studied naturally occurring non-flavonoid polyphenols from a group of stilbenes is resveratrol (3,5,4′-trihydroxystilbene; RSV) [129]. The positive effects of RSV in many diseases have been described as antidiabetic properties [130] and antimicrobial [131], antitumorigenic [132], antioxidant [133], neuroprotective [94,95] antiproliferative and anti-inflammatory action [134] via inhibition of prostaglandin synthesis and it also exhibits apoptosis. Resveratrol has structural and functional homology with estrogen and thus can bind to nuclear estrogen receptors (ER) and regulate their activity [135]. Aryl hydrocarbon receptor (AhR), essential for immune response as the mediator of dioxin toxicity which induces immunosuppression, can bind with estrogen receptors or NF-κB and lead to an inflammatory response [134]. This non/flavonoid polyphenol affects signal transduction pathways such as AKT, STAT3, RPS6KB2, MAPK, PKC, and PPAR-γ [134]. Resveratrol is a natural antagonist of AhR and can inhibit Th17 cells and also modulate suppression of NF-κB, IL-1β, COX, and LPS to decrease inflammation and ROS levels [134]. This phytoestrogen can modulate ovarian function (oocyte maturation) and steroidogenesis and can protect oocytes from senescence-dependent damage by activation of the sirtuin1 gene (SIRT1) [136]. Activation of the SIRT1 leads to the elevation of LH (luteinizing hormone) receptors and ovarian GnRH (gonadotropin-releasing hormone) receptors, stimulating mitochondria multiplicity and viability to enhance anti-oxidative capacity [137]. RSV can modulate the reduction of inflammatory gene expression as IGF-1 (insulin-like growth factor 1). While HGF (hepatocyte growth factor) are overexpressed in the peritoneal fluid of women with endometriosis, and it can effectively inhibit PGF-2α which induces uterine contraction, leads to vessel relaxation and thus improves blood flow and reduces ischemia [137]. Resveratrol could also be promising in endometriosis as well as uterine fibroids as it significantly reduces the number and volume of endometrial implants [138]. On the other hand, it has an anti-deciduogenic effect in the endometrium, and therefore resveratrol supplementation should be avoided during the luteal phase and pregnancy [139]. In PCOS patients, administration of resveratrol significantly decreased total testosterone [140]. Resveratrol added to the culture medium during in vitro cultivation of oocytes increased blastocyst formation, reduced OS of vitrified mice oocytes, and improved oocyte maturation and embryo development [141].

### 3.5. Trace Elements—Zinc and Selenium and Their Fertility Effects

Essential trace elements in the human body are zinc (Zn), selenium (Se), copper (Co), chromium (Cr), cobalt (Co), iodine (I), manganese (Mn), and molybdenum (Mo). These are most effective in active sites of enzymes or as a part of transcription factors [142]. A deficiency in these trace elements (e.g., Zn, Se) is commonly linked with reproduction disorders such as endometriosis, PCOS [143], and pregnancy disorders such as prolonged labor, or pregnancy-induced hypertension [144,145].

**Figure 5:**
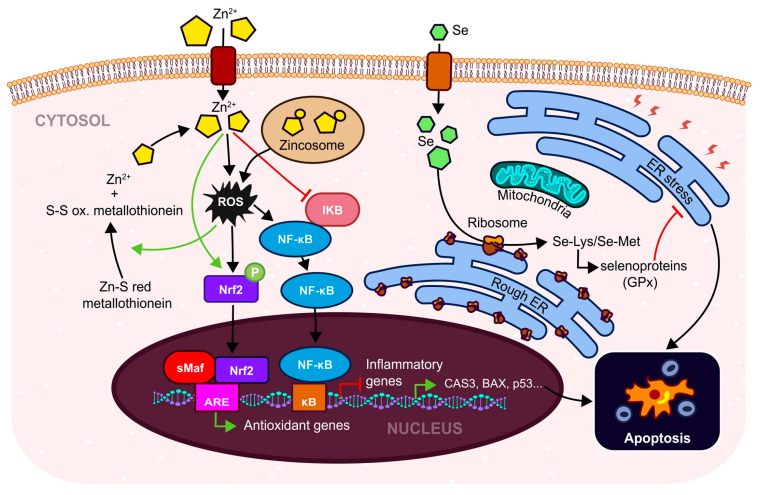
The action mechanism of zinc and selenium in response to oxidative stress. Zinc can be released from metallothionein under protein reduction (formation of disulfidic bond after releasing zinc bound to sulfur in its oxidized form). Free zinc can act directly in reducing OS or can act via activation of Nrf2 signaling (green arrows) and can inhibit (red line) NF-κB signaling. On the other hand, selenium is incorporated in the amino acids lysine and methionine, and is used in the proteosynthesis of antioxidant enzymes and selenoproteins such as GPx which have the ability to reduce oxidative stress (red line).

Zinc (in divalent form Zn^2+^) is the second most frequently occurring element in living organisms [51] and has an essential role in the regulation of several physiological processes of female germ cell growth, fertility, and pregnancy [143]. Zinc can occur in the cell in two forms, either bound with proteins, so-called zinc-finger proteins, or it may be present in association with some proteins in the cytoplasm, mitochondria, and secretory vesicles [51]. Zinc fulfils an important function in maintaining endocrine and redox balance, inflammatory processes, glucose, and lipid metabolism, and in regulating cell proliferation, gene expression, and the immune system [143]. The direct antioxidant effect of zinc is mediated via metallothionein (Figure 5) in which Zn^2+^ is bound to sulphur residue in a reduced-protein form that can be oxidized and which releases free Zn^2+^ [146,147]. Zinc can stabilize the Zn-finger domain of NF-κB [148] where it acts as an alternative inhibitor of IKB [149]. As a transcription regulator of Nrf2, it can upregulate some downstream antioxidants through nuclear translocation and thus respond to damage caused by ROS [150]. Zinc, together with copper, is a cofactor for one of the most important antioxidant enzymes, Zn-SOD/SOD1 [151], which has a crucial role in women’s ovulation and menstrual cycles by scavenging ROS [152]. Additionally, Zn itself is a regulator of the zinc-dependent extracellular matrix remodeling endopeptidases known as MMPs [108,109]. Matrix metalloproteinases are involved in endometrium shedding, restructuralization, and decidualization [153,154]. They are essential in the early development of the preimplantation embryo as well as in the development of the fetus [51] via enhancing the activity of the STAT3/MMPs axis [155] In the invasion and migration of embryonic cells. Also affect the neurodevelopment of brain cells [156,157]. During pregnancy, a lower concentration of zinc is linked to prolonged labor, pre-eclampsia, preterm birth, and post-term pregnancy [143]. Zinc impacts mitochondrial homeostasis via its antioxidant and prooxidant properties [148]. A low concentration of Zn^2+^ affects mitochondrial biogenesis as matrix-related MMPs require zinc for maturation of protein. Furthermore, zinc is also an inhibitor of respiratory complex IV, redox-dependent pathways in the mitochondria and is involved in endoplasmatic reticulum OS and protein misfolding [148].

Another micronutrient which is important for the proper functioning of the body in terms of immunity, nervous system, muscles, and reproductive health is selenium [158]. Selenium, in a similar way to zinc, can act as an antioxidant via selenomethionine or selenocysteine, where selenium is mainly converted into selenomethionine and selenocysteine and is incorporated into proteins in place of methionine/cysteine which is essential for the synthesis of all selenoproteins (Figure 5) such as PGX, TXNRD, SEPHS2, SELENOF, SELENOH, and SELENOM [159,160]. Selenium mediates the inhibition of NF-κB, expression of inflammatory cytokines [161,162], and improves the antioxidant capacity via activating Nrf2, thereby alleviating the cytotoxicity caused by ROS [163]. As a component of seleno-proteins such as GPx and thioredoxin reductase, selenium has a crucial role in redox re-actions [134] in the uterus, granulosa cells, and follicles [164]. In addition to antioxidant properties, selenium also has anti-inflammatory, anti-mutagenic, anti-carcinogenic, chemopreventive, and antimicrobial effects [165]. A reduced concentration of selenium in the blood of pregnant women is linked to a risk factor for subfertility [144] or can be linked to some defects in fetus development [166]. Selenium can suppress metritis and ovarian cysts [167]. A deficiency in Se is a cause of miscarriages and stillbirths [165]. The level of selenium decreases during pregnancy [168]. Insufficient selenium is also associated with decreased female fertility, probably due to its antioxidant role in the ovaries [168]. It has been proven that a lower level of selenium in follicular fluid is linked to unexplained infertility and premature ovarian failure in the use of the specific selenium-binding protein 1 [169]. Selenium from the follicular fluid is positively associated with embryo development, blastocyst formation, and embryos of high quality [170]. Selenium is potentially protective for trophoblast cells from excessive OS via the reduction of ROS through activating mitochondria biogenesis during the stimulation of trophoblast cells with selenium [171]. Some studies state that oral selenium supplementation improves embryo quality [172] and increases the number of good-quality oocytes in older women undergoing IVF therapy [173].

## 4. Conclusions

Antioxidants can counteract the damaging effects of oxidation in a living organism. The cellular antioxidant defense system protects cells from attack by ROS. There are three major types of ROS: superoxide radicals (O_2_^•−^), hydrogen peroxide (H_2_O_2_), and hydroxyl (^•^OH) radicals. Based on increased OS during the fertilization process and oocyte maturation, antioxidants are crucial for the proper functioning of female reproduction. Therefore, imbalance between excess of ROS and lack of antioxidants could negatively affect female fertility. In this review, the selected naturally occurring antioxidants exhibit improvement in the endometrial thickness of the uterine lining which is very important for the successful implantation of the embryo. Supplementing with antioxidants during the treatment of infertility might improve the outcome of assisted reproduction techniques. Some antioxidants are promising in preventing pre-eclampsia and preterm birth. The administration of antioxidants to patients with reproductive disorders such as PCOS, endometriosis, or functional hypothalamic amenorrhea improved the course of the diseases. However, not all antioxidants are recommended for pregnant women. Despite the positive effects of resveratrol and quercetin supplementation in gynecological diseases, it is recommended to avoid such polyphenols during pregnancy.

## Figures and Tables

**Table 1:** Recommended daily supplementation dose of some naturally occurring antioxidants for women in different cases (ARD mg/day).

Example	Adult	Pregnant	In ART	Role	Source
Vitamin A 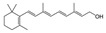	0.7–0.77 [39,40]	0.55–0.77 [40]	0.6–5.8 [41]	cell differentiation, antioxidant [34]	sweet potato, carrot, tuna, pumpkin, kale
Vitamin C 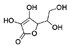	75–85 [39,40]	70–85 [40,42]	127–167 [41]	cofactor of oxidases, antioxidant, and wound healing process [43]	guavas, bell pepper, kiwifruit, broccoli, papaya, strawberries
Vitamin E 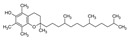	15 [39]	12–15 [40]	8.5–11 [41]	antioxidant, modulates signal transduction, cell division, and cell membrane integrity [44]	sunflower seeds, olive oil, almonds, avocado, spinach, butternut squash, kiwifruit
Vitamin B9 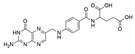	0.4–0.6 [39]	0.4 [45]	0.2–0.4 [41]	coenzyme in single carbon transferase, aids in the production of nucleic acids [43]	liver, yeast, legumes, pulses, fermented foods, and leafy vegetables [46,47]
Selenium	0.055–0.06 [39]	0.05–0.06 [48]	≤0.075 [49]	part of glutathione peroxidases, potent intracellular antioxidant enzymes [43]	Meat, fish, milk, eggs, cruciferous vegetables, liliaceus vegetables, legumes, garlic, onion [50]
Zinc	8–11 [39]	9.5–11 [40]	≤18.6 [51]	part of regulatory and catabolic enzymes responsible for signal transduction, gene expression structural role in zinc-finger motifs [43]	oysters, beef, chicken, firm tofu, squash and pumpkin seeds, low-fat yogurt, and lentils
L-carnitine 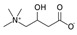	500 [52]	3000 [53]	1000 [54]	facilitates long-chain fatty acid entry into cellular mitochondria [55]	sheep meat, beef, pork, fish, chicken, cow’s milk
Melatonin 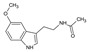	approx. 0.5–10 [56,57,58]	0.3–0.6 [41]	3–6 [54,59]	antioxidant [60], pro/apoptotic, regulates the uptake of growth factors, increases immunosurveillance, anti-angiogenic [61]	cherries, walnuts, mustard seeds, poppy seeds, corn, rice
Quercetin 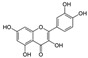	100–500 [62,63]	1000 [63]	1200 [64], 30/kg [65]	antioxidant, anti-inflammatory, antibacterial, antiviral [66], and inhibits cell proliferation [67]	fruits, vegetables, seeds, nuts
Resveratrol 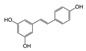	not specified	avoided [33]	60–120 [68]	antioxidant, modifies cell morphology, gene expression, ligand-receptor interactions, signaling pathways foam-cell formation [69], and modulates innate and adaptive immunity [70]	skins of grapes, red and white wines, apples, blueberries, peanuts

## Data Availability

Not applicable.
